# Denture Stomatitis Revisited: A Summary of Systematic Reviews in the Past Decade and Two Case Reports of Papillary Hyperplasia of Unusual Locations

**DOI:** 10.1155/2021/7338143

**Published:** 2021-10-13

**Authors:** Samiha Yousef Sartawi, Shaden Abu-Hammad, Nesreen A. Salim, Salah Al-Omoush

**Affiliations:** ^1^Faculty of Dentistry, University of Jordan, Amman 11942, Jordan; ^2^Fixed and Removable Prosthodontics Consultant, Jordan University Hospital, Amman, Jordan; ^3^University of Jordan, Amman, Jordan

## Abstract

**Objectives:**

Denture stomatitis is a mucosal condition associated with complete and partial removable dentures. This paper presents a short review of the literature on this topic with more emphasis on the treatment protocols of denture stomatitis as studied in recent systematic reviews.

**Methods:**

A general review of the literature was carried out in the first part of this paper, and then the most recent systematic reviews on the treatment protocols of denture stomatitis were summarized.

**Results:**

Fifteen systematic reviews were collected and classified into six main treatment protocols.

**Conclusions:**

Dentist knowledge of up-to-date treatment options of denture stomatitis will assist comprehensive treatment planning. However, the newer methods of denture disinfection need further studies before recommendation.

## 1. Introduction

Denture stomatitis, a common disorder affecting denture wearers, is characterized by inflammation and erythema of the oral mucosal areas covered by the denture [1]. Despite its commonality, the aetiology of denture stomatitis is not completely understood [1]. The prevalence of denture stomatitis ranges from 20 to 67% among denture wearers [1, 2]. This is explained by the increase in denture usage with age as a large number of denture wearers are dependent elderly [3]. Furthermore, removable dentures are known to decrease the flow of oxygen and saliva to the underlying tissue, producing a local environment that facilitates yeast overgrowth [4]. *Candida albicans* colonization was found solely on the fitting surface of the prosthesis or in association with bacteria [5]. *Candida albicans* is almost always linked to denture stomatitis although it is a commensal microorganism in the oral cavity of 45–65% of healthy individuals; however, the prevalence of *Candida albicans* increases up to 60–100% in denture wearers [4].

Denture stomatitis is commonly seen on the palatal mucosa of the upper jaw, is more prevalent in female patients, and is considered to be a benign lesion [2]. However, the mucosa under mandibular dentures is protected by salivary flow, so it is hardly affected [6]. Buccal and labial mucosa which are in direct contact with the denture base might exhibit denture stomatitis [5]. According to Newton's classification in 1962, type I denture stomatitis is where the mucosal inflammation is localised as a result of trauma. In Newton's types II and III denture stomatitis, the denture‐bearing mucosa is diffusely involved [2], and it is granular in type III. Inflammatory papillary hyperplasia, granular denture stomatitis, and Newton's type III are all common terminologies for the advanced stage of the condition [5]. The condition may not be painful but may cause some unfavourable symptoms such as burning or tingling sensation underneath the denture, and it might be associated with angular cheilitis, atrophic glossitis, acute pseudomembranous candidiasis, and chronic hyperplastic candidiasis [5, 7].

Denture stomatitis or *Candida*-induced denture stomatitis is a multifactorial condition [2]. There is an interaction between a number of predisposing local and systemic factors. Local risk factors which are associated with denture stomatitis are dry mouth, local trauma from an ill-fitting denture, poor denture hygiene, continuous denture wearing, carbohydrate-rich diets, and acidic salivary pH [8] in addition to smoking [9]. Poor oral hygiene and the continuous use of dentures were found to be the most significant risk factors for developing denture stomatitis [10]. Candidal growth has also been associated with dentures relined with the soft liner [5].

Systemic predisposing factors including dietary deficiency, immunosuppression, immunodeficiency, and haematological disorders may play a part by reducing the individual immunity to defeat the disease [8]. For example, Martorano-Fernandes et al. demonstrated a higher prevalence of denture stomatitis among diabetic patients who might be at a higher risk of developing *Candida* infections. However, they stated that the quality of the searched evidence was very low in their systematic review. The authors highlighted the importance of screening patients who were presented with oral candidiasis for diabetes [11]. Another study found that the female subjects in the stomatitis and control groups had lower vitamin D serum levels than their male counterparts which were linked to increased denture stomatitis severity [8]. Interestingly, salivary biomarkers were identified in patients with denture stomatitis as the palatal inflammation was significantly associated with the levels of salivary cytokines [12].

Management of most cases of denture stomatitis requires a comprehensive treatment plan initiated by identifying the predisposing factors [6]. Therefore, clinicians should always start with the elimination of denture faults, instructions on the control of denture plaque, and discontinuous denture wearing at night time [5]. However, there is a huge body of literature about different management protocols which is confusing to the general dentist in case he/she tries to search a treatment recipe. On the contrary, systematic reviews remain a strong source of evidence-based science which is not commonly read by general dentists in comparison to those in academia. Therefore, the aim of this paper is to collect the most valid information from up-to-date systematic reviews on the treatment of denture stomatitis in the last 10 years and to make it easier and faster for the dentist who is looking for precise and quick information.

Interestingly, two case reports were presented at the end of this paper. The authors aimed to link these two clinical cases to outline an uncommon presentation of denture stomatitis on areas other than the palatal mucosa. This will help the general practitioner expect these lesions on other areas in the oral cavity.

## 2. Treatment of Denture Stomatitis: A Summary of Systematic Reviews in the Last 10 Years

### 2.1. Materials and Methods

Online search was carried out in PubMed and Google Scholar (publication date from January 2010 to January 2021) with the key words “denture stomatitis,” “denture induced stomatitis,” “denture associated stomatitis,” and “systematic review.” The inclusion criteria were systematic reviews (1) written in English and (2) in the scope of denture stomatitis treatment. The exclusion criterion was the absence of focus on one or more treatment protocols.

For the case reports, ethical approval was sought from the organisation where the authors are based. Ethical approval was not required according to the protection of human subjects and animals in research where only the oral cavity is visible in clinical pictures and the subjects are unidentifiable. Consent was obtained from the subjects to include their clinical pictures in research and teaching.

## 3. Results

Eighteen systematic reviews matched the search criteria. Three papers were excluded because no specific treatment protocol was studied.  Table 1 represents the papers included in this study; the first three papers were excluded. The fifteen systematic reviews were classified according to the main treatment method.

### 3.1. Denture Liners and Tissue Conditioners as a Route for Treatment

Tissue conditioners are used to refresh the traumatised mucosa underlying ill-fitting dentures as a temporary treatment, while soft liners can be utilised for the same purpose for a longer period of time [28]. Regarding denture liners, Skupien et al. suggested that 0.5% sodium hypochlorite can help disinfect denture liners and tissue conditioners. Furthermore, the incorporation of 500.000 IU/mL nystatin to denture lining materials was able to treat or prevent oral candidiasis. Nevertheless, the authors claimed that there was insufficient evidence to recommend one ideal method to clean these materials [13]. Iqbal and Safar reported the efficacy of incorporating conventional organic antifungal agents (nystatin, azole group derivatives, and chlorhexidine), antimicrobials/antifungals other than organic (silver zeolite, silver nanoparticles, photocatalysts, and metallic oxides), and natural and herbal antimicrobials (tea tree oil, lemongrass essential oil, and origanum oil) into many tissue conditioners with minimal or no effects on physical and mechanical properties of the materials [15]. Shaikh et al. included 25 articles in their systematic review on the effects of adding nystatin to tissue conditioners. Their review reported that the addition of nystatin is beneficial, with minimal consequences, if not at all, on the mechanical and physical features of tissue conditioners. So, they suggested that adding nystatin to tissue conditioners could be a method for treating denture stomatitis but urged the need for more studies to determine the optimum nystatin dosage and release control mechanism plus the stability of tissue conditioners including these antifungals over a period of time [14].

### 3.2. Antifungal Topical Agents

While nystatin remains the standard treatment for mild oral candidiasis (Clinical Practice Guidelines for the Management of Candidiasis: 2009 update by the Infectious Diseases Society of America) [29], there is a vague knowledge in the literature regarding the administered dosage or pharmaceutical form, duration, and side effects. Lyu et al. included three clinical studies on the treatment of denture stomatitis. One of the studies showed that using nystatin pastilles for 2 weeks could achieve up to 28.6% clinical cure rate and up to 71.4% mycological cure rate. Another study administered the pastille form for 4 weeks which showed a 76.9% clinical cure rate and a 40% mycological cure rate [16]. Lyu et al. concluded that nystatin administration for 4 weeks has a better clinical efficacy than 2 weeks in the treatment of denture stomatitis. Dosage of 400,000 IU of nystatin pastilles showed a mycological effect which was significantly higher than that of the 200,000 IU pastilles. The studies also reported that using a dosage of 100,000 IU suspension form of nystatin was effective for a duration of 2 weeks and was able to achieve up to 53% clinical cure rate without mentioning the mycological effect. Other studies, included in the review, carried on cancer patients with oral candidiasis suggested that the combination of the suspension formula (100,000 IU) and pastilles (100,000 IU) for 2 weeks can be more effective than the suspension alone [16]. Whether this modality is applicable to denture stomatitis or not, it is still a question to be answered.

### 3.3. Disinfecting Agents

Emami et al. evaluated 14 randomized clinical trials and found no statistical significant difference between antifungal treatment methods and disinfecting agents for the treatment of denture stomatitis. They concluded that disinfection agents, antiseptic mouthwashes, natural substances with antimicrobial properties, microwave disinfection, and photodynamic therapy could be an adjunct or alternative to antifungal medications in the treatment of denture stomatitis [17]. Another systematic review assessed the level of evidence available on the effectiveness of any agent for treating or preventing denture stomatitis; Hilgert et al. included 35 randomized clinical trials and found a high risk of bias in 32 papers. The authors demonstrated that the use of nystatin and disinfecting agents in the treatment of denture stomatitis was well supported in the results; however, they warned the clinicians to be aware that the available studies had a high risk of bias and the overall quality of those reports was low [18].

### 3.4. Microwave, Photodynamic Therapy, and Laser Disinfection

Seven years after Khiyani et al. [12], Da Costa et al. evaluated five randomized clinical trials which applied microwave disinfection for the treatment of denture stomatitis. They stated that the treatment with 650 W for three minutes once a week for 14 days had better cost-effect results and was as effective as 0.2% chlorhexidine, 0.02% sodium hypochlorite, and topical nystatin (100.000 IU/mL) and superior to topical miconazole in treating denture stomatitis [19]. Santos Sousa et al. studied the effects of microwave disinfection and antifungal therapy on the *Candida* counts and clinical manifestation of denture stomatitis. They recommended microwave disinfection of complete dentures as an efficient antifungal therapy for the treatment of denture stomatitis. However, they urged further well-designed studies to confirm such evidence [20]. They reported a significant reduction in *Candida* counts and the frequency of denture stomatitis in the groups subjected to microwave disinfection and topical nystatin therapy for a 90-day follow-up period. Microwave irradiation has also been described as an alternative method of disinfection of denture lining materials [13]. Another systematic review found that microwave therapy, for denture wearers, was significantly better than topical miconazole treatment [21].

Photodynamic therapy is another way of denture disinfection. The procedure is based on using a photosensitizing agent which is activated by light of appropriate wavelength [30]. This interaction in the presence of oxygen produces reactive oxygen and free radicals, which cause *Candida* cell damage and death [31]. Kellesarian et al. studied the efficacy of photodynamic therapy in the disinfection of acrylic surfaces of microorganisms on acrylic specimens where one RCT found that photodynamic therapy was comparable to antifungal drugs for denture disinfection. However, the authors recommended that the behaviour of photodynamic therapy is still unclear, and there is a need for more controlled clinical studies [22]. Davoudi et al. studied low-level laser therapy (LLLT) in comparison to the other treatment regimens in a systematic review. LLLT was introduced as a mean of denture disinfection in four moderate strength scientific papers, and it has shown a significant role in the clinical treatment of denture stomatitis and has been successful in reducing the colony-forming unit (CFU/mL) of *Candida albicans* and alleviating soft tissue inflammation caused by denture stomatitis, without any significant side effects [23].

### 3.5. Denture Base Resins (DBRs)

Although many efforts have been made to improve the antimicrobial ability of denture base resins, An et al.'s systematic review found that the effectiveness of incorporating of antimicrobial agents into DBRs has not been demonstrated conclusively [24]. Verhaeghe et al. explored the effects of overnight storage of dentures in water on the colonization of *Candida* on the denture surface. Only four articles matched the inclusion criteria in that review. The authors concluded that cleaning dentures at night before overnight storage helps reduce *Candida albicans* colonization. If cleaning was not possible as in the case of institutionalized elderly, then the use of alkaline peroxide cleaning tablet should be considered. If the tablet is not available, overnight dry storage is an option for reducing *Candida albicans* colonization, with clinically insignificant changes to the dimensions of the complete denture. The authors showed that storing dentures in water alone may promote *Candida albicans* colonization if the dentures were not cleaned; however, they advised that the current evidence requires more studies [25].

### 3.6. Other Methods

Interestingly, Nair et al. explored the clinical effectiveness of *Aloe vera* in the management of different oral mucosal diseases in a systematic review. Only one study was performed on denture wearers where denture cleansing tablets containing triphala, *Aloe vera*, and cashew leaf were used to disinfect complete dentures of 50 patients. In that study, *Aloe vera* gel showed a significant reduction of *Candida* count compared to water [26]. Hu et al. analysed six randomized controlled clinical trials in their systematic review which was focused on the effects of probiotics in the treatment of oral candidiasis [27]. Probiotics showed favourable results in preventing and treating oral candidiasis in the elderly and denture wearers compared to the placebo and blank groups. However, more evidence is required to compare the safety and effectiveness of probiotics to conventional antifungal treatments [27].

The prevalence of inflammatory papillary hyperplasia was not exclusively studied in the literature because most authors fail to specify the stage of denture stomatitis in their studies; however, it was found to be 4.43% [10]. As mentioned previously, papillary hyperplasia is mostly seen on the upper jaw, especially the palate, and hardly seen on the lower jaw [6, 32]. Papillary hyperplasia has been associated with ill-fitting dentures and smoking [33]. Removable dentures could be either complete or partial dentures with full or partial palatal coverage [10]. Therefore, this paper sought to report two different locations of papillary hyperplasia other than the palatal mucosa.

### 3.7. Case Report 1

A 70-year-old male patient presented to the undergraduate removable prosthodontics clinic where the author is based, seeking a new set of dentures. The patient's chief complaint was that the current denture is broken and loose. This denture was 15 years old with no follow-up visits to the dentist after insertion. No symptoms or pain were experienced by the patient while using the denture. The patient was hypertensive and on oral medication (*β*-blockers). Blood pressure was under control.

**Figure 1:**
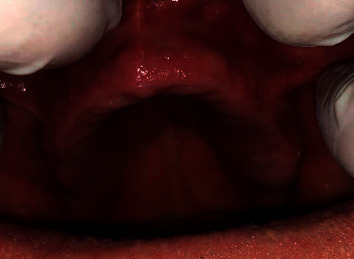
Upper edentulous ridge showing papillary nodules at the labial aspect of the ridge.

During extraoral examination, no abnormality was detected. Upon intraoral examination, there was diffuse red and inflamed oral mucosa on the palate in addition to multiple small papillary nodules at the labial surface of the premaxilla (Figure 1).

**Figure 2:**
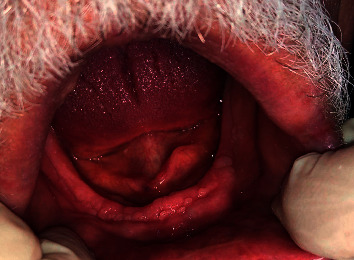
Lower edentulous ridge showing papillary nodules at the labial aspect of the ridge.

**Figure 3:**
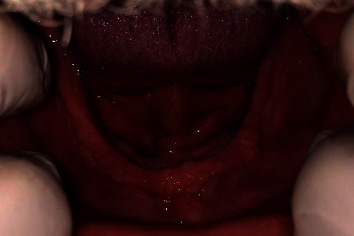
A closer view of the lower edentulous ridge showing papillary nodules at the labial aspect of the ridge.

Upon examination of the lower jaw, multiple nodular, well-rounded smooth mucosal proliferations were seen at the labial aspect and the left buccal shelf area on the lower edentulous ridge.  Figure 2 shows the papillary-like bodies at the lower ridge.  Figure 3 shows a closer view of the lower edentulous ridge with the papillary nodules at the labial aspect of the ridge. Palpation of these mucosal elevations (papillae) revealed soft mobile firmly attached structures with a normal mucosal outer layer. No ulcerations or changes in the colour of the mucosa were detected around or elsewhere in the oral cavity.

The patient was encouraged to discontinue wearing the old set of dentures and to use a nystatin mouthwash twice daily for two weeks. The diffuse redness on the palate and the papillary lesions on the premaxilla and the mandible faded after two weeks and the construction of a new denture were started.

### 3.8. Case Report 2

**Figure 4:**
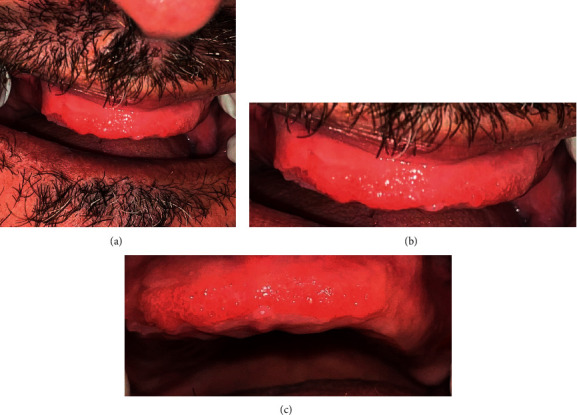
(a) The upper edentulous ridge of case 2 showing hyperplasia of the labial mucosa, (b) the same area in (a) zoomed in, and (c) the same area in (a, b) zoomed in.

A 75-year-old male patient presented to author's prosthodontics speciality clinic seeking the construction of a new denture. The chief complaint was that the lower denture is loose. Both upper and lower dentures were 25 years old, and the patient was satisfied with the upper denture. The patient was diabetic and hypertensive and consuming oral medications (Glucophage and concor); both medical conditions were well controlled. Figures 4(a)–4(c) show papillary proliferations on the labial mucosa of the premaxilla. These mucosal lesions are similar in appearance to class III Newton's denture stomatitis or papillary hyperplasia of the palate. Unfortunately, the patient failed to attend more clinics due to serious illness; therefore, no follow-up data were available.

## 4. Discussion

This paper represents a review of denture stomatitis, its aetiology, clinical presentation, and a summary of the recent systematic reviews regarding the treatment. It is clear that our basic knowledge has not changed much in the last decade with regard to the multifactorial nature of the condition and the need for a comprehensive management protocol to come over the risk factors or, if possible, to say the causative factors. The summary of the systematic reviews since 2010 was aimed so that the most recent critical appraisal of the scientific papers in the field is collected and reported together. This was thought to be helpful and handy for clinicians to find up-to-date information about this condition although for some interventions, the authors warned readers that the results were not conclusive, and more clinical research is required. This paper highlighted the most commonly used methods and their significance in the reduction of denture stomatitis. These methods were extracted from highly ranked scientific papers, i.e., the systematic reviews. It was interesting to find new approaches for the treatment of the fragile mucosa or the disinfection of the denture itself such as using probiotics [27], natural products [26], or laser [23].

One shocking fact was that keeping the dentures dry overnight is better than storing them in water uncleaned as the latter may promote *Candida albicans* colonization [25]. The authors also pointed out that keeping the dentures dry after cleaning will not affect their mechanical or physical properties. This may shift our approach in teaching undergraduate dental students and patients on the maintenance of the delivered complete dentures because cleaning at night and storing in water might be time-consuming and frustrating for some elderly patients.

Inclusion of the two case reports in this paper was supplementary overall. Each patient case is unique in the clinical presentation of denture stomatitis, especially speaking the denture-induced papillary hyperplasia. Histopathology: such lesions are described as mucosal projections covered by the stratified squamous epithelium with or without chronic inflammation with no premalignant components [10]. According to Newton's classification, this is considered as class III where the affected area is localised and papillary in texture [2]. In the first case, the papillae were seen as grape-like clusters on the crest of the lower edentulous arch and the labial mucosa. In the second case, the labial mucosa of the premaxilla was papillary in shape. It was stated that buccal and labial mucosa which are in direct contact with the denture base might exhibit denture stomatitis [5], but no further reports were found. Case reports are important because they bring attention to new interventions or clinical presentations of norms and pathology. The appearance of papillary hyperplasia on the mandible and on buccal mucosa is not uncommon because any area on the supporting mucosa could be traumatised from the continuous use of ill-fitting dentures for a long period of time. It has been postulated that wearing the same denture for a duration more than 10 years is the most significant risk factor to develop papillary hyperplasia on the foundation mucosa [34]. It is not fully understood why the palatal mucosa is mostly affected with most forms of denture stomatitis, but mostly related to the power of saliva cleansability underneath the lower denture. The increased surface area of the palate combined with the reduced access of saliva under the upper denture makes the palate a good candidate for denture-induced stomatitis.

The treatment of papillary hyperplasia is not different from denture stomatitis. However, the presence of tissue excess which might not heal with conservative treatment will require further aggressive management. Therefore, the treatment varies widely among clinicians according to the severity of the condition and the clinical presentation [19]. When the clinical presentation is aggressive laser excision, electrosurgery or cryotherapy is recommended [10]. Small localised lesions have been topically treated with chlorhexidine mouthwash [35]. In the first case report, the lesions on the mandible disappeared by not wearing the lower denture and using chlorhexidine mouthwash to cure the palatal erythema.

One limitation of this paper is that the summary of the literature is focused on the last decade (from 2010 to 2021). Systematic reviews after January 2021 were not included due to the preparation and submission of the manuscript. In addition to this, case reports provide excellent clinical examples on certain conditions or treatment plans. However, presenting only two case reports is considered a limitation of this study where conclusions cannot be withdrawn although both cases are useful examples.

## 5. Conclusion

Denture stomatitis is a multifactorial condition seen mostly in the upper jaw, especially on the palatal mucosa as mentioned before. The management of the condition must be comprehensive starting with proper diagnosis of the causative and risk factors and then directing the treatment toward the most significant factors which are patient specific. Practitioners can search for a quick source of information which is evidence-based and summarized under the same heading “Treatment of Denture Stomatitis.”

Another important conclusion is that papillary hyperplasia is not exclusively seen on the palate; it might be seen on other areas related to the upper or lower denture.

## Figures and Tables

**Table 1:** The systematic reviews obtained from the online search and the treatment method they evaluated.

No.	Authors	Title	Treatment method
1^*∗*^	Gual-Vaqués et al. [10]	Inflammatory papillary hyperplasia: a systematic review	No treatment method was reviewed

2^*∗*^	Martorano-Fernandes et al. [11]	Oral candidiasis and denture stomatitis in diabetic patients: systematic review and meta-analysis	No treatment method was reviewed

3^*∗*^	Khiyani et al. [12]	Salivary biomarkers in denture stomatitis: a systematic review	No treatment method was reviewed

4	Skupien et al.[13]	Prevention and treatment of Candida colonization on denture liners: a systematic review	Disinfection of the denture liner

5	Shaikh et al. [14]	Therapeutic role of nystatin added to tissue conditioners for treating denture-induced stomatitis: a systematic review	Nystatin

6	Iqbal and Zafar [15]	Role of antifungal medicaments added to tissue conditioners: a systematic review	Antifungal drugs

7	Lyu et al. [16]	Efficacy of nystatin for the treatment of oral candidiasis: a systematic review and meta-analysis	Nystatin

8	Emami et al. [17]	Linking evidence to treatment for denture stomatitis: a meta-analysis of randomized controlled trials	Disinfecting agents

9	Hilgert et al. [18]	Interventions for the management of denture stomatitis: a systematic review and meta‐analysis	Disinfecting agents and nystatin

10	Da Costa et al. [19]	The effectiveness of microwave disinfection in treating Candida-associated denture stomatitis: a systematic review and meta-analysis	Microwave disinfection

11	Santos Sousa et al. [20]	Effectiveness of denture microwave disinfection for treatment of denture stomatitis: a systematic review and meta‐analysis	Microwave disinfection

12	Zhang et al. [21]	Efficacy and safety of miconazole for oral candidiasis: a systematic review and meta‐analysis	Miconazole

13	Kellesarian et al. [22]	Efficacy of antimicrobial photodynamic therapy in the disinfection of acrylic denture surfaces: a systematic review	Photodynamic therapy

14	Davoudi et al. [23]	Role of laser or photodynamic therapy in treatment of denture stomatitis: a systematic review	Laser and photodynamic therapy

15	An et al. [24]	Incorporation of antimicrobial agents in denture base resin: a systematic review	Antimicrobial agents

16	Verhaeghe et al. [25]	The effect of overnight storage conditions on complete denture colonization by Candida albicans and dimensional stability: a systematic review	Denture base resins

17	Nair et al. [26]	Clinical effectiveness of aloe vera in the management of oral mucosal diseases—a systematic review	Aloe vera

18	Hu et al. [27]	In vivo effectiveness and safety of probiotics on prophylaxis and treatment of oral candidiasis: a systematic review and meta-analysis	Probiotics

^*∗*^Papers excluded from the summary.

## Data Availability

The data used to support the findings of this study are available from the corresponding author upon request.

## Additional Points

At the end of the paper, two case reports of uncommon locations of advanced denture stomatitis are presented.
